# Impact of the COVID-19 pandemic on human salmonellosis in the Netherlands

**DOI:** 10.1017/S0950268821002557

**Published:** 2021-12-02

**Authors:** Lapo Mughini-Gras, Linda Chanamé Pinedo, Roan Pijnacker, Maaike van den Beld, Ben Wit, Kees Veldman, Thijs Bosh, Eelco Franz

**Affiliations:** 1 Centre for Infectious Disease Control (CIb), National Institute for Public Health and the Environment (RIVM), Bilthoven, the Netherlands; 2 Institute for Risk Assessment Sciences (IRAS), Utrecht University, Utrecht, the Netherlands; 3 Netherlands Food and Consumer Product Safety Authority (NVWA), Utrecht, the Netherlands; 4 Wageningen Bioveterinary Research (WBVR), Lelystad, the Netherlands

**Keywords:** Control measures, COVID-19, impact assessment, *Salmonella*

## Abstract

The public health measures implemented to control coronavirus disease 2019 (COVID-19) may influence also other infectious diseases. Using national laboratory surveillance data, we assessed the impact of the COVID-19 pandemic on human salmonellosis in the Netherlands until March 2021. Salmonellosis incidence decreased significantly after March 2020: in the second, third and fourth quarters of 2020, and in the first quarter of 2021, the incidence decreased by 55%, 57%, 47% and 37%, respectively, compared to the same quarters of 2016–2019. The decrease was strongest among travel-related cases (94%, 84%, 79% and 93% in the aforementioned quarters, respectively). Other significant changes were: increased proportion of cases among older adults and increased proportion of invasive infections, decreased proportion of trimethoprim resistance and increased proportion of serovar Typhimurium monophasic variant *vs.* Enteritidis. This led to decreased contributions of laying hens and increased contributions of pigs and cattle as sources of human infections. The observed changes probably reflect a combination of reduced exposure to *Salmonella* due to restrictions on international travels and gatherings, closure of dine-in restaurants, catering and hospitality sectors at large and changes in healthcare-seeking and diagnostic behaviours.

The coronavirus disease 2019 (COVID-19) pandemic caused by the severe acute respiratory syndrome coronavirus 2 (SARS-CoV-2) has had a dramatic public health and socioeconomic impact globally, with several public health measures being implemented to control its spread. In the Netherlands, these measures have been implemented intermittently since mid-March 2020 and included social distancing, ‘stay-at-home’ and teleworking recommendations, closure of public spaces (restaurants, entertainment venues, non-essential shops, etc.) and educational institutions (day-care, schools, universities, etc.), restrictions on gatherings and international travels and use of protective masks in indoor public spaces and public transportation [1]. From mid-May 2020 to mid-October 2020, some measures were relaxed, including (partial) reopening of day-care and primary schools and reopening of most public spaces. This was followed by a period of increasingly stringent measures being implemented to contain a new COVID-19 upsurge (the ‘second wave’), which forced the country into a new lockdown from mid-December to March 2021.

Although these measures were intended to reduce SARS-CoV-2 transmission, they also had direct and/or indirect effects on the transmission of other infectious diseases. This has been described for respiratory tract infections other than COVID-19 [2, 3], vaccine-preventable diseases [1, 4], sexually transmitted infections [4] and to a lesser extent for gastrointestinal infections [4].

This study aimed to assess the impact of the COVID-19 pandemic in the Netherlands on a major gastrointestinal infection, salmonellosis, from January 2020 to March 2021. *Salmonella* infection is the second most reported zoonosis in Europe [5], where it usually causes self-limiting diarrhoeal illness with low case fatality [6]. Yet, *Salmonella* may sometimes invade beyond the intestine, causing invasive infections, which are being increasingly observed in the Netherlands [7]. When adjusting for underreporting, an estimated 27 000 symptomatic *Salmonella* infections occur annually in the Netherlands (~17 million population), ~70% of which caused by serovars Enteritidis and Typhimurium (including its monophasic variant) [8]. Salmonellosis incidence has decreased substantially since the mid-1990s in the Netherlands [8], with ~80% of human cases being attributable to pigs and laying hens as animal reservoirs. A concurrent decrease in egg-associated salmonellosis and an increase in pig- and reptile-associated salmonellosis have also been observed [8]. Our hypothesis is that the COVID-19 pandemic led to a decrease in human salmonellosis cases, mostly as a result of reduced exposure to *Salmonella* due to restrictions on international travels and gatherings (including house parties, barbecues, receptions, etc.), closure of dine-in restaurants, catering and the hospitality sectors at large and possibly changes in healthcare-seeking and diagnostic behaviours [4], as our society and the healthcare system have been put under pressure by the pandemic.

We used national surveillance data for 4788 serotyped *Salmonella* isolates from 4772 patients reported in the Netherlands during January 2016–March 2021. Different isolates from the same patient were selected only if they belonged to different serovars. The surveillance system is based on a laboratory network submitting clinical *Salmonella* isolates voluntarily to the National Institute for Public Health and the Environment (RIVM) for characterisation, with an estimated population coverage of 62% [7, 8]. Patient metadata for analysis were sex (female or male), age group (⩽4, 5–14, 15–59 or ⩾60 years), quarter (Q1–Q4) and year (2016–2021) of isolation (i.e. from Q1 of 2016 to Q1 of 2021), travel history during the incubation period (present or unknown) and type of infection (invasive or non-invasive infection, based on previous case definitions [7]). Antimicrobial resistance (AMR) profiling is performed on ~90% of the annual number of submitted isolates. The minimum inhibitory concentration for 14 antimicrobials was used to classify each isolate as resistant/susceptible based on epidemiological cut-offs of the European Committee on Antimicrobial Susceptibility Testing.

For source attribution analysis, we retrieved all serotyped *Salmonella* isolates from pigs (*n* = 248), cattle (*n* = 445), broiler chickens (*n* = 775), laying hens (*n* = 235) and reptile pets (*n* = 28) collected during 2016–2020 by the Dutch veterinary services (livestock) and private clinics (reptiles) as part of their routine activities on animals and foods. These non-human isolates are also submitted to the RIVM and analysed in the same way as the human isolates.

The incidence of salmonellosis reported in each quarter of 2020 and in Q1 2021 was compared with the incidence reported in the same quarters of 2016–2019 (pre-COVID-19 reference period) using a Poisson regression model. This model included the quarterly numbers of cases in the study period (from Q1 2016 to Q1 2021), stratified by age group and sex, as dependent variable, while the quarters under comparison, age group and sex were included as categorical independent variables. The respective yearly age group- and sex-specific number of residents in the Dutch population were included as offset variable. Estimates were expressed as incidence rate ratio and 95% confidence interval (95% CI). Subsequently, using line-list case data, five multivariable logistic regression models (one per quarter under study) were built to assess the quarterly differences in the proportions of cases with a known travel history, with an invasive infection, with an infection caused by the main serotypes (namely Enteritidis, Typhimurium and its monophasic variant, or others), or with an infection caused with isolates displaying resistance to the tested antimicrobials. The binary dependent variable was then being either a case reported in a given quarter under study, i.e. those in 2020–2021 (coded as ‘1’) or being a case reported in the corresponding quarters of the pre-COVID-19 reference period (coded as ‘0’), i.e. 2016–2019. Multi-collinearity among independent variables was checked using the variance inflation factor and selection between collinear variables was made based on an improved model fit (Akaike information criterion). A backward variable selection approach was then applied to retain only those variables showing significant associations with the outcome, i.e. variables for which the differences in their case distribution between the quarters under comparison were significant. Age and sex were always controlled for in the models. Estimates were expressed as odds ratio (OR) and 95% CI. A cluster–robust sandwich variance estimator was used to account for multiple isolates from a same patient. A *P*-value < 0.05 was considered statistically significant. All analyses were performed using Stata 16 (StataCorp).

Source attribution was performed using the modified Dutch model based on the 2016–2020 serotyping data, as described in detail previously [7, 8]. Briefly, the model infers probabilistically the sources of human cases by comparing their serovar distribution with that of the animal sources (i.e. pigs, cattle, broilers, layers and reptiles), weighted by the overall *Salmonella* prevalence in each source and the human exposure to them. Each year of human cases is attributed to 3 years of pooled data for pigs, cattle, broilers and layers and all years of reptile data, as done before [7, 8]. Differences in the attributable fractions between 2020 and 2016–2019 were tested with a two-sample test of proportions.

Figure 1 shows the quarterly incidences and Table 1 shows the Poisson regression results. Salmonellosis incidence in Q2, Q3 and Q4 of 2020, and in Q1 of 2021, was significantly lower than the incidence in the same quarters of 2016–2019, with overall reductions of 55%, 57%, 47% and 37%, respectively. No significant reduction was observed in Q1 of 2020.

**Fig. 1.**
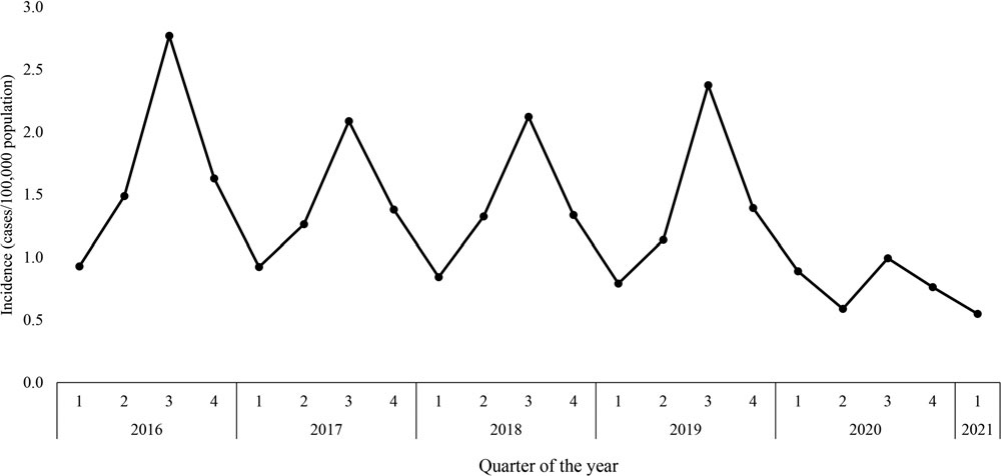
Quarterly salmonellosis incidence in the Netherlands, 2016–2021.

**Table 1.** Comparisons of salmonellosis incidence in the 2020 quarters and the first quarter of 2021 with the same quarters of the pre-COVID-19 reference period (2016–2019)

Quarter	2016–2019		2020		IRR	95% CI	*P*-value
	*N*^a^	Incidence^a^^,^^b^	*N*	Incidence^b^			
1	150	0.872	154	0.890	1.021	0.856–1.219	0.816
2	224	1.303	102	0.590	0.452	0.369–0.555	0.000
3	401	2.338	172	0.994	0.425	0.363–0.498	0.000
4	246	1.434	132	0.763	0.532	0.443–0.638	0.000
Quarter	2016–2019		2021		IRR	95% CI	*P*-value
	*N*^a^	Incidence^a^^,^^b^	*N*	Incidence^b^			
1	150	0.872	95	0.549	0.630	0.507–0.782	0.000

IRR, incidence rate ratio; 95% CI, 95% confidence interval.

Estimates are adjusted for age and sex.

a Average estimates over the years.

b Cases per 100 000 population.

The proportion of salmonellosis cases with a known travel history decreased significantly in Q2, Q3 and Q4 of 2020, and in Q1 of 2021, as compared to the same quarters of 2016–2019, with overall reductions of 94%, 84%, 79% and 93%, respectively (Table 2). Overall, the decrease in travel-related cases was responsible for 14%, 12%, 16% and 26% of the observed reduction in the total number of cases in Q2, Q3 and Q4 of 2020 and in Q1 of 2021, respectively. No significant reduction in travel-related cases was observed in Q1 of 2020.

**Table 2.** Comparisons of proportions of salmonellosis cases according to travel history, age group, sex, infecting serotype, invasiveness of infection and AMR in the 2020 quarters and the first quarter of 2021 with the same quarters of the pre-COVID-19 reference period (2016–2019)

	Q1 2020 (*n* = 156) *vs*. Q1 2016–2019 (*n* = 603)			Q2 2020 (*n* = 102) *vs*. Q2 2016–2019 (*n* = 904)			Q3 2020 (*n* = 173) *vs*. Q3 2016–2019 (*n* = 1629)			Q4 2020 (*n* = 133) *vs*. Q4 2016–2019 (*n* = 993)			Q1 2021 (*n* = 95) *vs*. Q1 2016–2019 (*n* = 603)		
	P%	C%	OR (95% CI)	P%	C%	OR (95% CI)	P%	C%	OR (95% CI)	P%	C%	OR (95% CI)	P%	C%	OR (95% CI)
Age group (years)															
0–4	9.95	6.41	0.72 (0.34–1.53)	8.52	6.86	0.63 (0.27–1.48)	8.84	13.29	1.02 (0.60–1.76)	9.97	10.53	1.08 (0.56–2.08)	9.95	10.53	0.77 (0.35–1.68)
5–14	10.45	14.74	1.58 (0.88–2.83)	11.73	10.78	0.69 (0.34–1.41)	15.22	12.72	0.57 (0.33–0.97)*	9.57	8.27	0.83 (0.41–1.68)	10.45	9.47	0.68 (0.31–1.50)
15–59	48.59	51.28	1.19 (0.78–1.80)	52.65	45.10	0.72 (0.45–1.14)	56.66	44.51	0.58 (0.40–0.85)**	49.75	42.11	0.83 (0.55–1.26)	48.59	37.89	0.65 (0.40–1.07)
>60	31.01	27.56	Ref.	27.10	37.25	Ref.	19.28	29.48	Ref.	30.72	39.10	Ref.	31.01	42.11	Ref.
Sex (male *vs*. female)	44.28	42.95	0.98 (0.68–1.41)	45.58	52.94	1.27 (0.83–1.94)	47.45	50.29	1.10 (0.80–1.52)	47.03	54.14	1.23 (0.85–1.78)	44.28	57.89	1.72 (1.10–2.70)**
Travel history (yes *vs*. unknown)			NS	15.93	0.98	0.06 (0.01–0.47)**	17.37	2.89	0.16 (0.07–0.40)***	13.49	3.01	0.21 (0.07–0.57)**	14.59	1.05	0.07 (0.01–0.47)**
Serotype															
*Enteritidis*			Ref.	29.87	19.61	Ref.	14.73	16.18	Ref.			Ref.			Ref.
*Typhimurium*			NS	17.92	13.73	0.97 (0.47–2.00)	13.26	21.39	1.30 (0.79–2.13)			NS			NS
Monophasic			NS	13.05	32.35	3.13 (1.71–5.72)*	33.33	29.48	1.74 (1.10–2.76)*			NS			NS
Other			NS	39.16	34.31	1.30 (0.72–2.36)	38.67	32.95	1.07 (0.71–1.60)			NS			NS
Trimethoprim resistance (yes *vs*. no)			NS			NS	7.24	2.31	0.28 (0.10–0.80)*			NS			NS
Infection type (invasive *vs*. non-invasive)			NS			NS			NS	6.45	15.79	2.57 (1.50–4.41)**			NS

OR, odds ratio; 95% CI, 95% confidence interval.

Estimates are adjusted for age and sex. P%, percentage of salmonellosis cases in a given category falling within the pre-COVID-19 reference period (2016–2019). C%, percentage of salmonellosis cases in a given category falling within the quarter under study during the COVID-19 period (Q1–Q4 2020 and Q1 2021). Ref., reference category. NS, not statistically significant and therefore not included in the model.

*P < 0.05, **P < 0.01, ***P < 0.001.

The proportion of cases with invasive salmonellosis was significantly higher in Q4 of 2020 (OR 2.57) than that in Q4 of 2016–2019, but no significant differences in invasiveness were observed in the other quarters (Table 2). The proportion of cases caused by the monophasic variant of *S*. Typhimurium *vs. S*. Enteritidis increased significantly in Q2 (OR 3.13) and in Q3 (OR 1.74) of 2020 as compared to the same quarters in 2016–2019. No other significant differences were observed with respect to the serovars.

Only for one antimicrobial, a significant difference was observed, i.e. the proportion of trimethoprim-resistant isolates was significantly lower in Q3 of 2020 (OR 0.28) than that in Q3 of 2016–2019. In Q3 of 2020 compared to Q3 of 2016–2019, the proportion of salmonellosis cases among the age groups 15–59 and 5–14 years was significantly lower (OR 0.58 and 0.57, respectively) than that in the elderly (>60 years), while the proportion of male *vs.* female cases was significantly higher (OR 1.72) in Q1 of 2021 than that in Q1 of 2016–2019 (Table 2).

The 564 human *Salmonella* isolates of 2020 were attributed to sources as follows: 38% to pigs, 23% to laying hens (i.e. eggs), 9% to cattle, 7% to broiler chickens and 6% to reptile pets, while 13% and 5% of cases were outbreak- and travel-related, respectively. As observed before, the proportion of cases attributable to travel decreased significantly, from 14–23% in 2016–2019 to 5% in 2020 (*P*-value < 0.001). Conversely, the contributions of pigs and cattle to human cases in 2020 increased significantly by, on average, 54% and 26% (both *P*-values < 0.001), respectively, as compared to 2016–2019. The contribution of laying hens decreased significantly by 17% (*P*-value = 0.025), thereby reflecting the increased occurrence of Typhimurium monophasic variant and the decreased occurrence of Enteritidis in 2020.

The incidence of reported salmonellosis in the Netherlands decreased significantly after the implementation of COVID-19 pandemic control measures in March 2020. Indeed, the decrease was significant in Q2–Q4 of 2020 and in Q1 of 2021, but not in Q1 of 2020, when everything was still ‘business as usual’. The decrease was particularly pronounced among cases with a known travel history, which normally account for ~20% of cases, but after Q1 of 2020 the travel-related cases decreased more than fourfold. A potential reason for this decrease is, therefore, reduced exposure to *Salmonella* as a result of travel restrictions, as only travels for essential purposes were permitted and even afterwards people were generally discouraged to travel. The trends observed in serovar distribution reflected the decrease in travel-related cases, as infection with *S*. Enteritidis in the Netherlands is more often associated with foreign travel (16–19% in 2016–2019) than infection with *S*. Typhimurium and its monophasic variant (4–8%) [9]. Also the attribution estimates reflected the changes observed in serovar distribution, as *S*. Enteritidis is a poultry-adapted serovar that is strongly associated with laying hens, and *S*. Typhimurium and its monophasic variant are more associated with pigs and cattle [8]. The few differences observed for AMR are, however, less straightforward to interpret and an explanation could be, once again, the drop in infections acquired abroad, as well as the changes in the relative contributions of the main sources of infection.

The observed decrease among non-travel-related cases was likely due to restrictions for both public and private gatherings, including those where food and drinks are normally served and might provide opportunities for large-scale exposure to *Salmonella*, such as receptions, parties, festivals, etc. Moreover, even if take-away and food delivery services have been active, the shutdown of dine-in services at restaurants, pubs, cafés and bars, including catering services, inevitably reduced the exposure to *Salmonella* via (contaminated) food consumed outside the household. This is a considerable source of infection as exemplified by one of the largest international outbreaks of *Salmonella* ever documented in Europe that has been recently investigated and microbiologically confirmed as linked to eating (products containing Polish eggs) in food establishments across 18 countries [10].

Our results do not exclude the possibility of altered healthcare-seeking behaviour, testing policy, diagnostic capacity and reporting compliance as additional factors contributing to the decreased salmonellosis incidence. Indeed, the healthcare system was overwhelmed by COVID-19 and strict triage procedures were enforced to assign priority to (severe) patients [1]. Therefore, a larger-than-usual number of salmonellosis cases with only mild to moderate symptoms could have been unascertained and unreported. Moreover, even the patients themselves could have refrained from seeking medical attention (to avoid contagion, reduce burden on healthcare, etc.). Some indications that this could have been the case were provided by our analysis. Indeed, in Q4 of 2020, when the second lockdown in the Netherlands started, there was a significant increase in the proportion of salmonellosis cases with invasive (i.e. extra-intestinal) infection, which are usually more severe and can be life-threatening due to bacteraemia, sepsis and infection of normally sterile sites [7]. This suggests that cases with more severe clinical manifestations might have been more likely to seek medical help, be attended by healthcare providers and therefore be ascertained and reported. Furthermore, an increase in salmonellosis cases among the elderly was also observed, which further supports the hypothesis of differential healthcare-seeking behaviour and possibly patient prioritisation depending on age, as the elderly are a high-risk group for COVID-19.

Regarding the observed age effect, however, alternative hypotheses can also be formulated. For example, because of the pandemic, the elderly might have found themselves more isolated and lonely and could have been less cared for (foodwise and hygienically) by relatives and friends. Moreover, it is conceivable that also the travel restrictions might have influenced the age distribution of cases, as younger people might be more likely to travel to high-risk destinations abroad. Finally, the proportion of travel-related cases is likely to be underestimated in the whole dataset. Indeed, because there is no ‘negative reporting’ when a case does not travel, a history of foreign travel is only reported when is known, but this information is not recorded systematically. Consequently, some cases with an unknown travel history might well be travel-related and, therefore, a larger proportion of salmonellosis reduction might be due to a decrease in cases with an unknown travel history that are actually travel-related.

In conclusion, the COVID-19 pandemic response had a significant impact on salmonellosis in the Netherlands, with drastically reduced incidence, especially among travel-related cases, as well as changes in age groups at highest risk, types of infection, AMR, serovar distribution and putative sources of infection. It is difficult to determine which factors contributed the most to these changes and it is likely that the underlying drivers are truly multifactorial, meaning that the observed changes are the result of a combination of reduced exposure to *Salmonella* through the typical pathways and changes in healthcare-seeking and diagnostic behaviours.

## Data Availability

All data relevant to the study are included in the article in an aggregated and anonymised format. For legal reasons, the disaggregated dataset is available in an anonymised format from the corresponding author on reasonable request.
